# Supplementary material for: Inflammatory Biomarkers in the Diagnosis and Prognosis of Rheumatoid Arthritis–Associated Interstitial Lung Disease
Source: Int J Mol Sci. 2023 Apr 5;24(7):6800. doi: 10.3390/ijms24076800 (PMC10095191; doi:10.3390/ijms24076800)
Supplement: Supplementary file 1 [file ijms-24-06800-s001.zip › ijms-2288746-supplementary.pdf]

International Journal of *Molecular Sciences*

MDPI

Article

Inflammatory biomarkers in the diagnosis and prognosis of rheumatoid arthritis–associated interstitial lung disease

Natalia Mena-Vázquez [1,2,*], Francisco Javier Godoy-Navarrete [3], Jose Manuel Lisbona-Montañez [1,2,4], Rocío Redondo-Rodriguez [1,2], Sara Manrique-Arija [1,2,4], José Rioja [1,4], Arkaitz Mucientes [1,2], Patricia Ruiz-Limón [1,5,6], Aimara Garcia-Studer [1,2], Fernando Ortiz-Márquez [1,2], Begoña Oliver-Martos [7], Laura Cano-García [1,2] and Antonio Fernández-Nebro [1,2,4].

[1] Instituto de Investigación Biomédica de Málaga (IBIMA)-Plataforma Bionand, 29010 Málaga, Spain
[2] UGC de Reumatología, Hospital Regional Universitario de Málaga, 29009 Málaga, Spain
[3] UGC de Reumatología, Hospital Universitario de Jaén, 23007 Jaén, Spain; fjgodoynavarrete@gmail.com
[4] Departamento de Medicina y Dermatología, Universidad de Málaga, 29010, Málaga, Spain
[5] UGC de Endocrinología y Nutrición, Hospital Clínico Virgen de la Victoria, 29010 Málaga, Spain
[6] CIBER Fisiopatología de la Obesidad y Nutrición (CIBEROBN), Instituto de Salud Carlos III, 28029 Madrid, Spain
[7] UGC de Neurociencias, Hospital Regional Universitario de Málaga, 29010 Málaga, Spain
* Correspondence: nataliamenavazquez@gmail.com
† These authors share first authorship

Citation: Mena-Vázquez, N.; Godoy-Navarrete, F.J.; Lisbona-Montañez, J.M.; Redondo-Rodriguez, R.; Manrique-Arija, S.; Rioja, J.; Mucientes, A.; Ruiz-Limón, P.; Garcia-Studer, A.; Ortiz-Márquez, F.; et al. Inflammatory Biomarkers in the Diagnosis and Prognosis of Rheumatoid Arthritis–Associated Interstitial Lung Disease. *Int. J. Mol. Sci.* **2023**, *24*, 6800. https://doi.org/10.3390/ijms24076800

Academic Editors: Catalin Codreanu and Elena Rezus

Received: 2 March 2023
Revised: 22 March 2023
Accepted: 3 April 2023
Published: 5 April 2023

Copyright: © 2023 by the authors. Licensee MDPI, Basel, Switzerland. This article is an open access article distributed under the terms and conditions of the Creative Commons Attribution (CC BY) license (https://creativecommons.org/licenses/by/4.0/).

Table S1. Progress of lung disease at the end of follow-up in 35 patients with RA-ILD.

Variable	Baseline	End of follow-up	p Value
Duration of follow-up, months, mean (SD)	-	66.1 (47.2)	-
Pulmonary function tests			
FVC predicted (%), mean (SD)	69.4 (14.8)	63.0 (17.1)	0.001
FVC <80%, n (%)	22 (62.9)	28 (80.0)	0.093
FVC ≥80%, n (%)	13 (37.1)	7 (20.0)	
FEV1 predicted (%), mean (SD)	75.1 (13.8)	68.7 (15.9)	0.003
DLCO-SB predicted (%), mean (SD)	68.9 (14.4)	61.0 (15.2)	<0.001
Radiologic progression, HRCT			-
Worsening, n (%)	-	9 (25.7)	
Stabilization, n (%)		24 (68.5)	
Improvement, n (%)		1 (2.8)	
Pulmonary function tests*			-
Improvement, n (%)	-	2 (5.7)	
Stabilization, n (%)		20 (57.1)	
Progression, n (%)		13 (37.1)	

Abbreviations. RA: rheumatoid arthritis; ILD: interstitial lung disease; SD: standard deviation; FVC: forced vital capacity; FEV_1: forced expiratory volume in the first second; DLCO: diffusing capacity of the lung for carbon monoxide; HRCT: high-resolution computed tomography; *Total lung outcome: progression was defined as worsening of FVC >10% or DLCO >15%, nonprogression as stabilization or improvement of FVC ≤10% or DLCO ≤15%, and improvement as an increase in FVC >10% or DLCO >15%.

Table S2. Baseline characteristics of 35 patients with RA-ILD and progression of lung disease.

VARIABLE	RA-ILD with progression N = 13	RA-ILD without progression N = 22	p Value
Epidemiologic characteristics			
Age, years, mean (SD)	69.6 (9.2)	69.8 (9.6)	0.949
Male sex, n (%)	8 (61.5)	7 (31.8)	0.086
Clinical-analytical characteristics			
Smoking history			0.845
Nonsmokers, n (%)	6 (46.2)	10 (45.5)	
Ex-smokers, n (%)	4 (30.8)	7 (31.8)	
Active smokers, n (%)	3 (23.1)	5 (22.7)	
Duration of RA, months, median (IQR)	180.7 (108.0-254.7)	137.6 (79.5-244.6)	0.511
Duration of ILD, months, mean (SD)	83.3 (40.0)	59.0 (49.0)	0.100
RF+ (>10 IU), n (%)	13 (100.0)	19 (86.4)	0.263
High RF (>60 IU)	10 (76.9)	14 (63.6)	0.413
ACPA+ (>20 IU), n (%)	13 (100.0)	19 (86.4)	0.164
High ACPA titer (>340 IU), n (%)	11 (84.6)	11 (50.0)	0.041
Radiographic erosions, n (%)	8 (61.6)	13 (59.0)	0.660
Clinical manifestations			
DAS28-ESR, mean (SD)	3.2 (1.0)	3.1 (0.9)	0.602
Remission-low disease activity, n (%)	6 (46.2)	13 (59.1)	0.347
Moderate-high disease activity, n (%)	7 (53.8)	9 (40.9)	0.347
HAQ, mean (SD)	1.3 (0.7)	1.2 (0.6)	0.511

Current treatment			
csDMARD, n (%)	11 (84.6)	17 (77.3)	0.600
Methotrexate, n (%)	8 (61.5)	11 (50.0)	0.508
Leflunomide, n (%)	0 (0.0)	3 (13.6)	0.164
Sulfasalazine, n (%)	2 (15.4)	0 (0.0)	0.058
Hydroxychloroquine, n (%)	2 (15.4)	4 (18.2)	0.832
Mycophenolate, n (%)	2 (15.4)	2 (15.4)	0.572
bDMARD, n (%)	8 (61.5)	14 (63.6)	0.901
Anti-TNF, n (%)	2 (15.4)	2 (15.4)	0.572
Tocilizumab, n (%)	2 (15.4)	1 (4.5)	0.268
Abatacept, n (%)	4 (30.8)	9 (40.9)	0.549
Rituximab, n (%)	0 (0.0)	2 (9.1)	0.263
Corticosteroids, n (%)	7 (53.8)	15 (68.2)	0.396
Dose of corticosteroids, mg/d mean (SD)	6.6 (2.8)	7.9 (4.0)	0.649
Pulmonary function testing			
FVC <80%, n (%)	13 (100.0)	15 (68.2)	0.031
FVC mean (SD)	56.1 (17.3)	67.3 (15.8)	0.056
FEV1 <80%, n (%)	10 (76.9)	13 (60.0)	0.201
FEV1 mean (SD)	64.3 (17.1)	71.5 (14.9)	0.207
DLCO <80%, n (%)	13 (100.0)	16 (72.7)	0.039
DLCO, mean (SD)	62.9 (13.9)	56.6 (17.7)	0.049
HCRT radiological pattern			
UIP, n (%)	12 (92.3)	17 (77.3)	0.254
NSIP, n (%)	1 (7.7)	5 (22.7)	0.254

Abbreviations. RA: rheumatoid arthritis; ILD: interstitial lung disease; SD: standard deviation; RF: rheumatoid factor; ACPA: anti–citrullinated peptide antibodies; csDMARD: conventional synthetic disease-modifying antirheumatic drug; bDMARD: biologic disease-modifying antirheumatic drug; FVC: forced vital capacity; FEV1: forced expiratory volume in the first second; DLCO: diffusing capacity of the lung for carbon monoxide; HRCT: high-resolution computed tomography; UIP: usual interstitial pneumonia; NSIP: nonspecific interstitial pneumonia.

Table S3. Cox regression model of factors associated with progression of RA-ILD.

Dependent variable	Predictor	HR	95% CI	p Value
Progression of ILD*				
	IL-18, pg/mL	1.229	1.026-1.472	0.025

*Progression was defined as worsening of FVC >10% or DLCO >15%, nonprogression as stabilization or improvement in FVC ≤10% or DLCO ≤15%, and improvement as increase in FVC >10% or in DLCO >15%.

Abbreviations: RA: rheumatoid arthritis; ILD: interstitial lung disease; IL: interleukin.

Variables included in the equation: age, sex, high ACPA, IL-1 alpha, IL-18, MCP-1/CCL4.
